# High-resolution dendrometer measurements reveal different responses of Douglas-fir to extreme drought in 2018 depending on soil and rooting characteristics

**DOI:** 10.3389/fpls.2024.1485440

**Published:** 2024-11-26

**Authors:** Göran Spangenberg, Reiner Zimmermann, Manfred Küppers, Sebastian Hein

**Affiliations:** ^1^ Department of Silviculture, University of Applied Forest Sciences Rottenburg, Rottenburg am Neckar, Germany; ^2^ Forest Ecology and Remote Sensing Group, Department 190a, Institute of Biology, University of Hohenheim, Stuttgart, Germany; ^3^ Department 190a, Institute of Biology, University of Hohenheim, Stuttgart, Germany

**Keywords:** non-native tree species, *Pseudotsuga menziesii*, extreme drought stress, soil texture, rooting characteristics, dendrometer, tree water deficit, growth duration

## Abstract

**Introduction:**

Douglas-fir (*Pseudotsuga menziesii* (Mirb.) Franco) is considered an important non-native substitute tree species in Europe, especially for Norway spruce (*Picea abies* (L.) Karst.), mainly due to its higher drought tolerance. However, Douglas-fir has also shown increasing mortality in certain regions of the world. One of the main reasons is the increase in drought and heat periods due to climate change. There is still a need for research on the influence of important soil properties and rooting characteristics on the drought tolerance of Douglas-fir. Therefore, we analyzed the influence of soil texture, plant-available water capacity (PAWC), fine root density, and effective rooting depth on water status and thus drought stress in Douglas-fir during the extreme drought of 2018.

**Methods:**

We selected seven closely spaced sites along a soil texture gradient from sand to clay at an elevation of ca. 500 m a.s.l. in southern Germany and determined soil physical and rooting characteristics. Water status parameters and growth duration were derived from dendrometer data at five Douglas-firs per site. The influence of soil and rooting characteristics on these drought stress-related parameters was analyzed using mixed-effects models. The focus was on two summer drought periods in 2018.

**Results and discussion:**

In the initial stage of the extreme summer drought of 2018 (in June), a higher PAWC and a higher fine root density reduced drought stress. However, these influences were no longer noticeable in the later stage of drought (in August), probably due to deeper soil desiccation. In August, a higher effective rooting depth reduced drought stress. Soil texture had a significant influence, particularly on growth duration. This study provides information on site selection for Douglas-fir cultivation under the predicted increase in severe drought, showing the importance of deep and intensive rooting, and points to the need for combined above- and belowground investigations for a better understanding of the drought response patterns of tree species.

## 1 Introduction

The climate change-induced increase in drought and heat events is causing massive damage to trees and tree mortality in many regions of the world (Hartmann et al., 2022; IPCC, 2023). For example, large parts of Central Europe were affected by extreme drought in 2018, which persisted in some areas until 2020 (Rakovec et al., 2022). In Germany alone, these extreme drought years caused a total volume of damaged wood of approximately 178 million m³ (Bolte et al., 2021). The most common tree species in Germany, namely Norway spruce (*Picea abies* (L.) Karst.), European beech (*Fagus sylvatica* L.), Scots pine (*Pinus sylvestris* L.), sessile oak (*Quercus petraea* (Mattuschka) Liebl.), and pedunculate oak (*Quercus robur* L.), were all affected by the extreme drought in 2018, although to varying extents (Schuldt et al., 2020). The reforestation of damaged forest areas and the adaptation of forest stands with a high drought risk are necessary on a huge scale (Bolte et al., 2021). Drought tolerance is one of the most important characteristics of tree species in a changing climate.

Douglas-fir (*Pseudotsuga menziesii* (Mirb.) Franco), a non-native tree species in Europe, is considered an important substitute for Norway spruce, which is widespread and economically important in Europe (Spiecker et al., 2019; Nicolescu et al., 2023). Douglas-fir has been assessed as more drought-tolerant and less climate-sensitive than Norway spruce, which shows high mortality under the current conditions of climate change and pronounced negative growth responses to drought-related weather extremes (Vitali et al., 2017; Schuldt et al., 2020; Nicolescu et al., 2023). Douglas-fir originated from a large area in western North America and was introduced to Europe in the 19th century. In Europe, Douglas-fir is currently most widespread in the western and central European countries of the Netherlands, Belgium, Luxembourg, France, and Germany. In these countries, Douglas-fir covers 2–5% of the national forest area (van Loo and Dobrowolska, 2019).

With an increase in extreme drought and heat periods, Douglas-fir in certain regions has been shown to be more sensitive to drought, with increased mortality. In the Klamath Mountains ecoregion of Oregon (USA), Bennett et al. (2023) showed that hot and dry sites were particularly affected by an increase in Douglas-fir mortality. Kane et al. (2014) studied Douglas-fir mortality in high-elevation mixed conifer forests in the southwestern United States following a severe drought. In France, increased damage and mortality were observed in Douglas-fir after the severe summer drought of 2003 (Sergent et al., 2014a, 2014b). A higher sensitivity to drought stress was found in Europe, specifically at lower elevations (Vejpustková and Čihák, 2019; Nicolescu et al., 2023). Summer drought is considered the greatest risk for Douglas-fir in Europe (Bastien, 2019). Increased mortality is also related to changes in the distribution of Douglas-fir in its natural area of origin (Littell et al., 2010; Flower et al., 2013; Bastien, 2019). If drought risk increases, site factors become increasingly important for the assessment of drought tolerance.

However, there is still a need for research on site factors that are important for assessing the drought risks of Douglas-fir, particularly with regard to soil texture and plant-available water capacity (PAWC). Soil texture largely determines the extent of soil water storage capacity and rooting (Köstler et al., 1968; Curt et al., 2001; Kutschera and Lichtenegger, 2013; Lwila et al., 2021). There are hardly any systematic studies covering a range of soil textures that determine the influence of soil texture on the drought tolerance of Douglas-fir. A study based on tree ring widths showed a higher risk of drought stress for clayey and certain silty sites (Spangenberg et al., 2024). PAWC is also a crucial site factor. It characterizes the water content between field capacity and wilting point, thus indicating the size of the water reservoir that trees can use (Veihmeyer and Hendrickson, 1927; Silva et al., 2014; Cousin et al., 2022). The few previous studies on the influence of PAWC on the drought tolerance of Douglas-fir have shown different results. Sergent et al. (2014b) found partially positive influences of a higher PAWC. In contrast, Huang et al. (2017) showed no clear influences, and Spangenberg et al. (2024) even found a reduction in drought resilience due to a higher PAWC.

Rooting is another factor closely linked to soil texture, and it is essential for drought tolerance. The depth and density of rooting have a direct influence on drought tolerance because roots are the trees’ access to soil water reserves (Bréda et al., 2006). The influence of rooting depth or root water uptake depth on drought vulnerability has been observed in different tree species (Nardini et al., 2016; Kahmen et al., 2022). There are very few studies on these relationships in Douglas-fir. Spangenberg et al. (2024) found that a higher rooting depth increased drought resilience. Rooting characteristics are important for our understanding of the growth responses of trees to drought and for adaptation processes (Lwila et al., 2023).

The following questions about Douglas-fir remained unanswered in the cited studies: Are there differences in the influence of site and rooting characteristics on the drought tolerance of Douglas-fir between the initial stage and the later stage of an extreme drought? What impact does drought period timing have? Does drought stress lead to a shorter duration of radial stem growth, and if so, what influence do site and rooting characteristics have? To investigate these questions, intra-annual measurement methods, such as temporally and spatially high-resolution measurements of stem radius changes (SRCs), are required. Dendrometers are used for these non-destructive measurements (Drew and Downes, 2009). The SRCs determined in this method are mainly based on irreversible stem growth and reversible processes (Deslauriers et al., 2007; Oberhuber et al., 2015b). Frost can lead to pronounced reversible stem thickness changes (Zweifel and Häsler, 2000). There is also a daily reversible variation in stem radius, which is mainly the result of differences in water potential between living bark tissue and xylem vessels (Daudet et al., 2005). Particularly on days with higher transpiration, water loss in the tree crown is faster than water uptake by the roots. This water loss can be partially or completely compensated for only during the following night. These differences in water potential result in hydration and dehydration processes in elastic tissues, mainly in the bark (Zweifel et al., 2000, 2001). Conclusions about daily water status can be derived from these SRCs. For this purpose, two stem water status parameters were derived from dendrometer data in this study: maximum daily shrinkage (MDS) and tree water deficit (TWD), also called tree water deficit-induced stem shrinkage (e.g., Köcher et al., 2013; Oberhuber et al., 2015a; Zweifel et al., 2016; Dietrich et al., 2018; Leštianska et al., 2020). MDS is a measure of the daily shrinkage of the stem that results from the daily hydration and dehydration processes described. This parameter characterizes the current plasticity and thus the ability of the tree to respond to actual weather and soil conditions (Giovannelli et al., 2007; Vieira et al., 2013; Güney et al., 2020). When evaluating MDS as a parameter for drought stress, the current weather conditions and possible water deficits already existing in the tree must also be considered (Zweifel et al., 2000; Güney et al., 2020). In contrast, TWD is a measure of water loss accumulated over several days if there is insufficient opportunity for water uptake (Drew et al., 2011; Oberhuber et al., 2015b; Zweifel et al., 2016). TWD is considered an indicator of the stem water deficit and thus of drought stress (Zweifel et al., 2005; Drew et al., 2011; Oberhuber et al., 2015b; Dietrich et al., 2018). The higher the TWD, the greater the drought stress. We also used dendrometer data to determine the onset, cessation, and duration of growth for the extreme drought year 2018 and for two reference years (2017 and 2019). Premature drought-induced growth cessation and thus a shortened growth duration can indicate drought stress. For example, trees with abrupt growth cessation or reductions show higher mortality rate (Das et al., 2007; Moran et al., 2017).

This study aimed to analyze the influences of the extreme drought year 2018 on TWD, MDS, and growth duration of Douglas-fir depending on two site characteristics (soil texture and PAWC) and two rooting characteristics (rooting depth and fine root density). For this purpose, a soil texture gradient from sand to clay was studied. We investigated a similar aim at the same study sites as in Spangenberg et al. (2024), but with a different methodology. At this first study, the influence of severe to extreme drought years in 2003/04 and 2018 on interannual radial growth (tree ring widths) and the growth response indices derived from it (resistance, recovery, resilience) were investigated. Here, we analyzed the influence of site and rooting characteristics on temporally high-resolution water status parameters TWD and MDS during two pronounced drought periods during the extreme drought in summer 2018, considering the temporal position of the drought period. We also investigated whether drought stress led to a shortening of the growth period and the influence of site and rooting characteristics. The present study contributes to a better understanding of the drought tolerance of Douglas-fir, depending on soil conditions and tree rooting characteristics. It provides important information for forest practices in the context of climate change, as these characteristics are relevant for deriving site-based silvicultural management recommendations.

After a comparison of the timing of stem growth onset and cessation from 2017 to 2019, which was used to assess the impact of the extreme drought in 2018, the following hypotheses were tested on Douglas-fir for the year 2018:

1. Water status parameters TWD and MDS show lower drought response on sandy, loamy, and silty soils compared to clayey soils during prolonged summer drought periods.
2. TWD and MDS show lower drought response with decreasing PAWC and increasing rooting depth during prolonged summer drought periods, while fine root density has no influence.
3. A higher TWD and MDS shorten the growth duration.
4. Soil texture, PAWC, and rooting depth have a significant influence on growth duration in the extreme drought year.

Hypotheses 1, 2, and 4 were formulated based on the results of Spangenberg et al. (2024).

## 2 Materials and methods

### 2.1 Study sites

Seven sites that differed in soil types and textures (1x sand-, 1x loam-, 3x silt-, 2x clay-dominated) and water balance (PAWC gradient from 44 to 101 mm) were evaluated (**Table 1**). These sites are located in southern Germany (Baden-Württemberg, 48°27’N, 8°58’E) in close proximity to each other (within a radius of 1 km). They are located at an elevation of 500–520 m a.s.l. and represent different sites and soils typical for the mountain range of the Triassic “Keuperbergland”. Due to the close proximity of the study sites and the comparable elevation, there are practically no, or at most minimal, differences in weather conditions. The area is characterized by a warm, temperate climate with warm summers and no recurrent dry season (Rubel et al., 2017). The average annual temperature from 1991 to 2020 was 9.3°C, recorded at the nearest weather station of the German Meteorological Service (Hechingen, elevation 517.5 m a.s.l.), with an average annual precipitation of 806.4 mm (DWD, 2021).

**Table 1:** Soil and rooting characteristics of the study sites.

Site abbreviation^a^	Sand	Loam	Silt1	Silt2	Silt3	Clay1	Clay2
Sites^b^							
Soil type (classification according to WRB)	Cambisol (epidystric, endoskeletic)	Planosol (albic, ruptic, epidystric)	Stagnic cutanic Luvisol (ruptic, epidystric, endosiltic)	Cambisol (endoeutric, ruptic)	Cutanic Luvisol (ruptic, hyperdystric, episiltic)	Vertic Cambisol (eutric, ruptic, epiclayic)	Vertic stagnic Cambisol (eutric, ruptic)
*Main soil texture*	*Sand*	*Loam*	*Silt*	*Silt*	*Silt*	*Clay*	*Clay*
Soil textures	0–40 cm: loamy sand deeper than 40 cm: sandstone slabs (parent material)	0–41 cm: sandy loam 41–100 cm: silt clay loam (parent material)	0–40 cm: silt loam 40–70 cm: silt clay loam 70–100 cm: clay (parent material)	0–41 cm: loam 41–100 cm: silt loam (parent material)	0–38 cm: silt loam 38–100 cm: silt clay loam	0–20 cm: loam 20–75 cm: clay 75–100 cm: clay (parent material)	0–34 cm: loam 34–82 cm: clay 82–100 cm: clay (parent material)
*PAWC (mm)*	*50*	*88*	*67*	*101*	*85*	*44*	*90*
PAWC-level	Very low	Low	Low	Medium	Low	Very low	Low
*Effective rooting depth (cm)*	*30*	*65*	*50*	*95*	*45*	*35*	*50*
*Fine root density (n/dm^2^)*	*6.8*	*10.0*	*4.7*	*14.6*	*6.4*	*11.1*	*9.2*
Relief							
Relief	Plain	SE-upper slope	Plain	NNE-middle slope	NNE-middle slope	N-middle slope	SE-lower slope
Slope (°)	2	7	3	9	6	12	5
Aspect (°)	343	143	4	24	20	354	144

The four predictors whose influence on the water status parameters and growth duration in the extreme drought year of 2018 were investigated are written in italics.

^a^Site abbreviations according to main soil texture for soil depth from 0 to 1 m **^b^** WRB: World Reference Base for Soil Resources (IUSS Working Group WRB, 2015), soil textures according to FAO (2006), main soil texture in the range of 0–1 m soil depth, PAWC: plant-available water capacity, PAWC-level according to Arbeitskreis Standortskartierung (2016), effective rooting depth: up to the threshold of three fine roots per dm^2^ (Arbeitskreis Standortskartierung, 2016), mean fine root density in 0–40 cm soil depth.

One soil profile was dug at each of the seven sites to determine the soil and rooting characteristics. To ensure that the results of root counting were comparable between sites, each soil profile was placed according to Arbeitskreis Standortskartierung (2016) so that the profile wall was located at the boundary between the outer and the middle third of the crown radius of a Douglas-fir. Fine and coarse roots were counted at the profile wall using 5 × 5 cm counting squares. Roots with a diameter < 2 mm were classified as fine roots (Böhm, 1979). For further calculations, values for two rooting characteristics were derived from the results of root counting: effective rooting depth and mean fine root density in a 0–40 cm soil depth (**Table 1**). A threshold of three fine roots per dm^2^ was used for the definition of effective rooting depth, in accordance with Arbeitskreis Standortskartierung (2016).

In addition, soil texture, skeletal and humus content, and bulk density were determined in the soil profile for each soil horizon up to a depth of 1 m, in accordance with Arbeitskreis Standortskartierung (2016) and Ad-hoc-AG Boden (2005). From these soil physical properties, PAWC (mm) was calculated for each soil horizon according to Ad-hoc-AG Boden (2005). For this, PAWC values were used based on samples of the forest soil survey in Baden-Württemberg (Puhlmann and von Wilpert, 2011; Arbeitskreis Standortskartierung, 2016) and were therefore well adapted to the local conditions. The total PAWC for the respective site was calculated down to the effective rooting depth using the PAWC values from the different soil horizons and considering the thickness of the horizons (Arbeitskreis Standortskartierung, 2016). Information on the superordinate soil texture was attributed to soil depths of 0–1 m (sand, silt, clay, and loam). The soil type of each soil profile was also determined and classified according to the World Reference Base for Soil Resources (IUSS Working Group WRB, 2015).

### 2.2 Meteorological data and delimitation of drought periods

Meteorological data [air temperature (°C), precipitation (mm)] were measured every half hour at a height of 2 m. For this purpose, a WatchDog 2700 weather station (Spectrum Technologies Inc., Plainfield, IL, USA) was placed in an open, unshaded area in the immediate vicinity of the study sites Sand, Silt1, Silt2, and Clay1.

**Figure 1:**
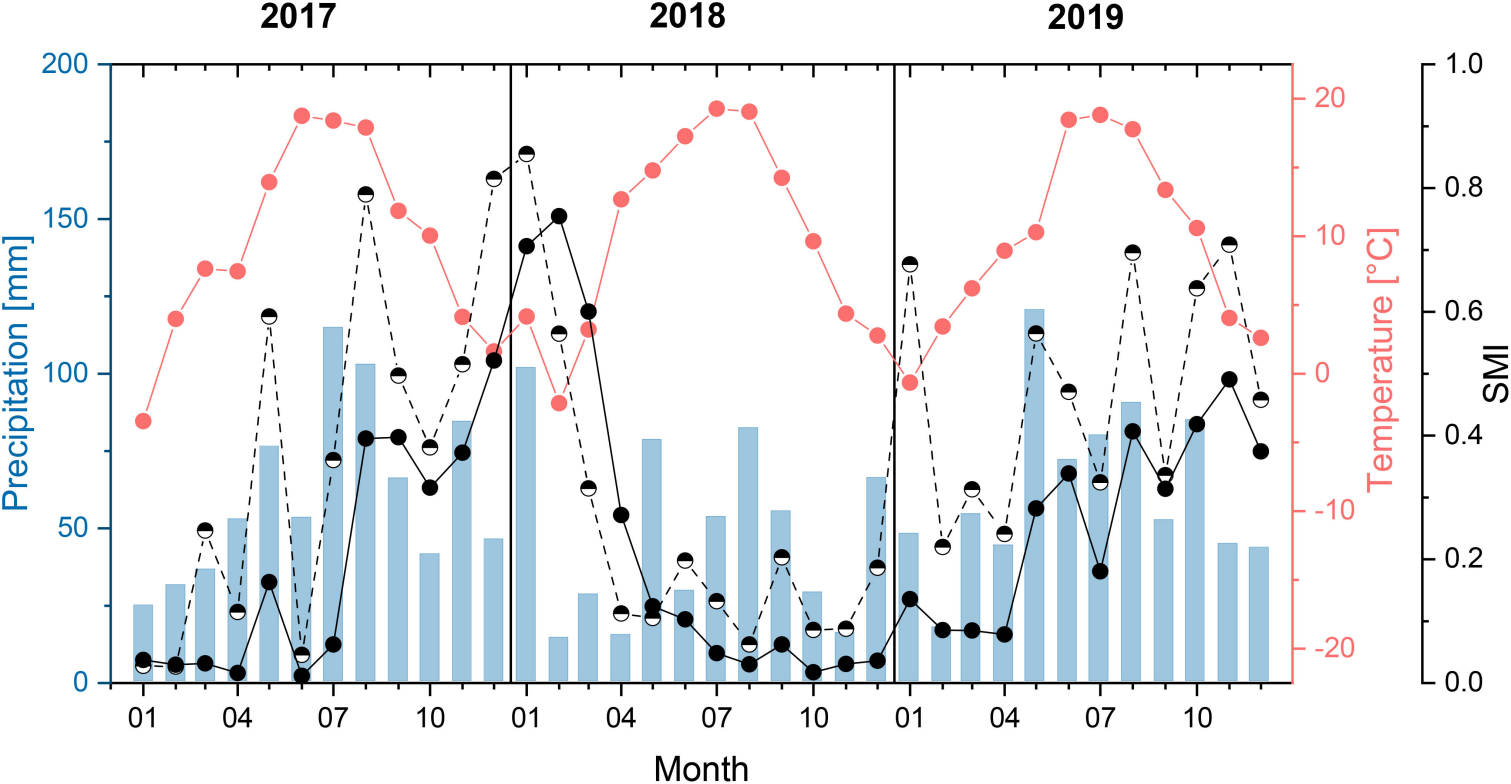
Monthly average temperature and precipitation sum (measured next to a study site) and soil moisture index (SMI, calculated grid data, provided by UFZ (2021). Half-filled circles = topsoil (uppermost 25 cm) and completely filled = total soil down to approximately 180 cm) for the study years and area.

To investigate the influences of site and rooting during a prolonged drought in the growing season (hypotheses 1 and 2), a main growing season with a prolonged drought (i.e., several months of drought) was identified. For this purpose, the soil moisture index (SMI) was analyzed, which was determined and provided for Germany by the Helmholtz Centre for Environmental Research–UFZ (UFZ, 2021). SMI was determined based on meteorological data from the German Meteorological Service as input parameter for soil moisture calculations via the mesoscale hydrologic model (MHM) (Samaniego et al., 2010). In this process, the local soil moisture was estimated for the entire root zone considering hydrological processes, such as interception, soil water dynamics, groundwater recharge, and storage (Zink et al., 2016). SMI represented the percentile of the simulated soil moisture value (moving average of the preceding 30 days) compared to 60-year soil moisture reconstruction (1954–2013) (Zink et al., 2016). Values vary between 0 and 1, and an increasing SMI indicates a decrease in drought (Samaniego et al., 2013). The SMI values we used were interpolated grid data for the topsoil (uppermost 25 cm) and total soil (down to approximately 180 cm, depending on soil properties) for the area of our study sites (Samaniego et al., 2013; Zink et al., 2016; UFZ, 2021). May–August 2018 proved to be a period with a pronounced drought. In all 4 months of this main growing season, the SMI for both the topsoil and the total soil was below the drought threshold of 0.2 (**Figure 1**). From May to August 2018, the SMI for the total soil decreased continuously and was below the threshold value of 0.05 in July and August, meaning that extreme drought occurred in these 2 months.

The next step was to identify particular drought periods lasting many days within the main growing season of 2018. There was no standard definition of a drought period (Slette et al., 2019). A frequently used meteorological definition based on precipitation defines a drought period as a certain minimum number of consecutive dry days. There are different precipitation thresholds for defining dry days, depending on the region and research question. In this study, the threshold values were defined so that TWD increased over the course of the drought period. We considered an occasional small amount of precipitation reaching the ground and thus a temporary slight decrease in TWD for a few days to be acceptable if the TWD generally increased over the entire drought period. The threshold value also considered interception losses due to the canopy. The canopy water storage capacity for different Douglas-fir forests was determined to be between approximately 1 and 4 mm (Pypker et al., 2005). Based on the age and structure of the stands, we assumed that canopy water storage capacity was no more than 3 mm for our study stands (Klaassen et al., 1998; Pypker et al., 2005; Andreasen et al., 2023; Spittlehouse and Maloney, 2023). However, the resulting interception losses were often higher due to evaporation of the intercepted water (Spittlehouse and Maloney, 2023).

Two complementary threshold values were used in this study to define the drought periods within the summer of 2018: (i) A threshold of 5 mm was applied for the maximum daily precipitation, as also used by König and Mayer (1989) for a drought index for the investigation of climatically induced forest damage. (ii) The average daily precipitation during the drought period was below 1 mm per day and thus below the assumed evapotranspiration in the summer (Jassal et al., 2009; Thomas et al., 2022). These two threshold values ensured that only consecutive dry days were selected as drought periods on which no or only a small amount of precipitation reached the soil and that the tree water deficit generally increased over the course of the drought period. The duration of the respective drought periods was also considered when selecting the studied drought periods. According to our hypotheses, we selected prolonged drought periods that caused a high level of drought stress for trees and reduced the influence of random effects, such as site-dependent differences in surface runoff during short intense rainfall events.

Based on these selection criteria and on the data from our weather station, two periods were identified as pronounced drought periods for the extremely dry summer of 2018. The first drought period lasted from June 13 to July 4 (in the following referred to as the “June drought period”, total precipitation during these 22 days amounted to 2.0 mm), and the second drought period occurred from August 8 to August 30 (“August drought period”, total precipitation during these 23 days was 16.6 mm, daily precipitation on each day < 5 mm). There was a stronger precipitation event directly before and after both drought periods, in which more than 15 mm of precipitation occurred within less than one day. TWD and MDS data starting from the morning after the end of the stronger precipitation event before the drought period were included in the analyses of the two drought periods. At this time, after the rain, the nightly replenishment of water storage tissues is complete (TWD usually achieves its minimum), and the contraction phase, which is decisive for calculating MDS, has not yet started. The TWD and MDS data included in the analysis of the drought periods ended with the onset of precipitation at the end of the drought period. This point in time was always clear due to a sudden stronger rainfall.

### 2.3 Stand and tree selection and genetic analysis

The stands at the investigated sites were Douglas-fir stands or in three cases mixed Douglas-fir and Norway spruce stands. While Norway spruce was spatially separated at sites Clay1 and Clay2, Douglas-fir was mixed with Norway spruce individually or in groups at the Silt3 site. Understory beech was mainly present at Clay2 and Loam sites. At each of the seven study sites, five Douglas-firs were selected per site for the dendrometer measurements (“dendrometer trees”). These trees had a social tree class of 1 or 2 (predominant or dominant trees) (Kraft, 1884) and had been selected as crop trees during previous thinnings. To minimize possible small site differences in the soil, the representativeness of the tree sites was examined for each tree using soil coring. The age of the trees was determined by tree ring coring and was approximately 40–55 years at the end of 2020 [with increment borers (Haglöf, Sweden)] (**Table 2**). The diameter at breast height (DBH) was determined, and the tree height and height of the crown base were measured with a Vertex (Haglöf, Sweden) for each dendrometer tree. Furthermore, stand characteristics were determined (stand density, stand basal area, DBH, and tree height refer to the tree with quadratic mean diameter), based on a DBH-full inventory of a representative stand area for each site (**Table 2**). We also derived the yield classes for Douglas-fir for each site from the growth tables for Baden-Württemberg (Forstliche Versuchs- und Forschungsanstalt BW, 2001). Input parameters were the measured heights and the tree age (determined from the number of years derived from the cores plus 5 years, as cores were collected at 1.3 m).

**Table 2:** Tree and stand characteristics of the study sites.

Site abbreviation	Sand	Loam	Silt1	Silt2	Silt3	Clay1	Clay2
Characteristics of the dendrometer trees (Douglas-firs)^a^							
DBH_Tree_ (cm)	52.4 (0.13)	36.2 (0.09)	49.3 (0.16)	56.9 (0.16)	62.3 (0.24)	36.6 (0.24)	34.5 (0.19)
H_Tree_ (m)	31.7 (0.73)	25.4 (0.94)	29.6 (0.55)	33.2 (0.33)	35.8 (0.36)	28.0 (1.02)	25.4 (0.64)
Crown length_Tree_ (m)	17.6 (0.77)	14.5 (0.98)	17.8 (0.68)	19.4 (0.50)	20.7 (0.73)	14.5 (0.69)	14.1 (0.53)
Age_Tree_, based on cores	42	35	40	42	49	42	37
Stand characteristics^b^							
N (ha^-1^)	326	840	379	392	300	416	770
BA (m^2^ ha^-1^)	26.0	29.5	35.5	33.0	35.8	26.6	29.8
DBH_g_ DF (cm)	41.4	32.8	41.3	49.7	56.1	36.0	28.7
H_g_ DF (m)	26.7	24.6	26.6	31.6	33.5	25.5	24.0
Yield class DF (m^3^ a^-1^ ha^-1^)	18-19	17	17-18	19	19	16	17

a DBH, stem diameter at breast height; H, tree height; Crown length, length of green tree crown; Crown base, height of the first living primary branch; values for these characteristics: arithmetic means (in brackets: standard error SE), based on measurement 3/2021; Age, number of years derived from the oldest annual tree ring per site until the end of 2020 determined by tree ring coring (actual tree age is higher due to sampling at breast height).

b N or BA, number of trees or basal area per hectare; DBHg (diameter at breast height) and Hg (tree height) refer to the tree with quadratic mean diameter; Yield class, mean total volume production per year up to age 100, based on Forstliche Versuchs- und Forschungsanstalt BW (2001); DF, Douglas-fir.

As the origin of a Douglas-fir tree may have a significant influence on drought tolerance (Eilmann et al., 2013; Jansen et al., 2013; Bansal et al., 2016; Chauvin et al., 2019), a genetic analysis was carried out for each of the five dendrometer trees per site. The cambium samples we collected for this purpose were genetically analyzed in the molecular genetic laboratory of the Institute of Silviculture at BOKU University in Vienna (Austria). The methods used were previously described in Slavov et al. (2004); van Loo et al. (2015); Hintsteiner et al. (2018); Neophytou (2019), and Spangenberg et al. (2024). As a result, all dendrometer trees were clearly assigned to the coastal variety of Douglas-fir (*Pseudotsuga menziesii* (Mirb.) Franco var. *menziesii*). The Douglas-fir trees of the seven study sites were genetically very similar at the 13 microsatellite loci analyzed, as no significant genetic differentiation was detected among them (Neophytou, 2019). Therefore, Douglas-fir trees from the seven study sites were pooled for all subsequent analyses.

### 2.4 Dendrometer measurements and derived parameters

Dendrometer trees were equipped with a high-resolution point dendrometer at a height of 1.6 m in March 2017. We used spring-loaded linear displacement potentiometers (type MMR 10_11 R5K, MEGATRON Elektronik GmbH & Co.KG, Munich, Germany) with analogue stepless resolution. Stem radius changes (SRCs) from 2017 to 2019 were recorded every half hour using a data logger (DL2e, Delta-T Devices Ltd., Cambridge, UK; DL18, Ecomatic, Dachau, Germany). Before mounting the dendrometers, as much as possible of the outermost dead bark layer was removed at the point where the contact head of the dendrometer touched the bark, without damaging the inner living cortex and cambium (Zweifel et al., 2006). The compensation or minimization of the possible weather-related sensitivity of the dendrometers (Zweifel et al., 2006) was carried out metrologically and mechanically. For example, the maximum resistance was simultaneously measured, and dendrometers were protected from rain and sunlight with a cover.

Raw SRC data were checked carefully for each tree. Clearly incorrect measurements (e.g., due to cable damage) were omitted. Due to a data logger defect in spring 2018, the data of site Clay2 could be used for evaluating drought periods but not for growth duration analysis. Characteristic values needed for further analyses and figures (e.g., daily maximum values, daily precipitation sum) were determined from SRC and weather data using the R package “dendrometeR” (van der Maaten et al., 2016).

**Figure 2:**
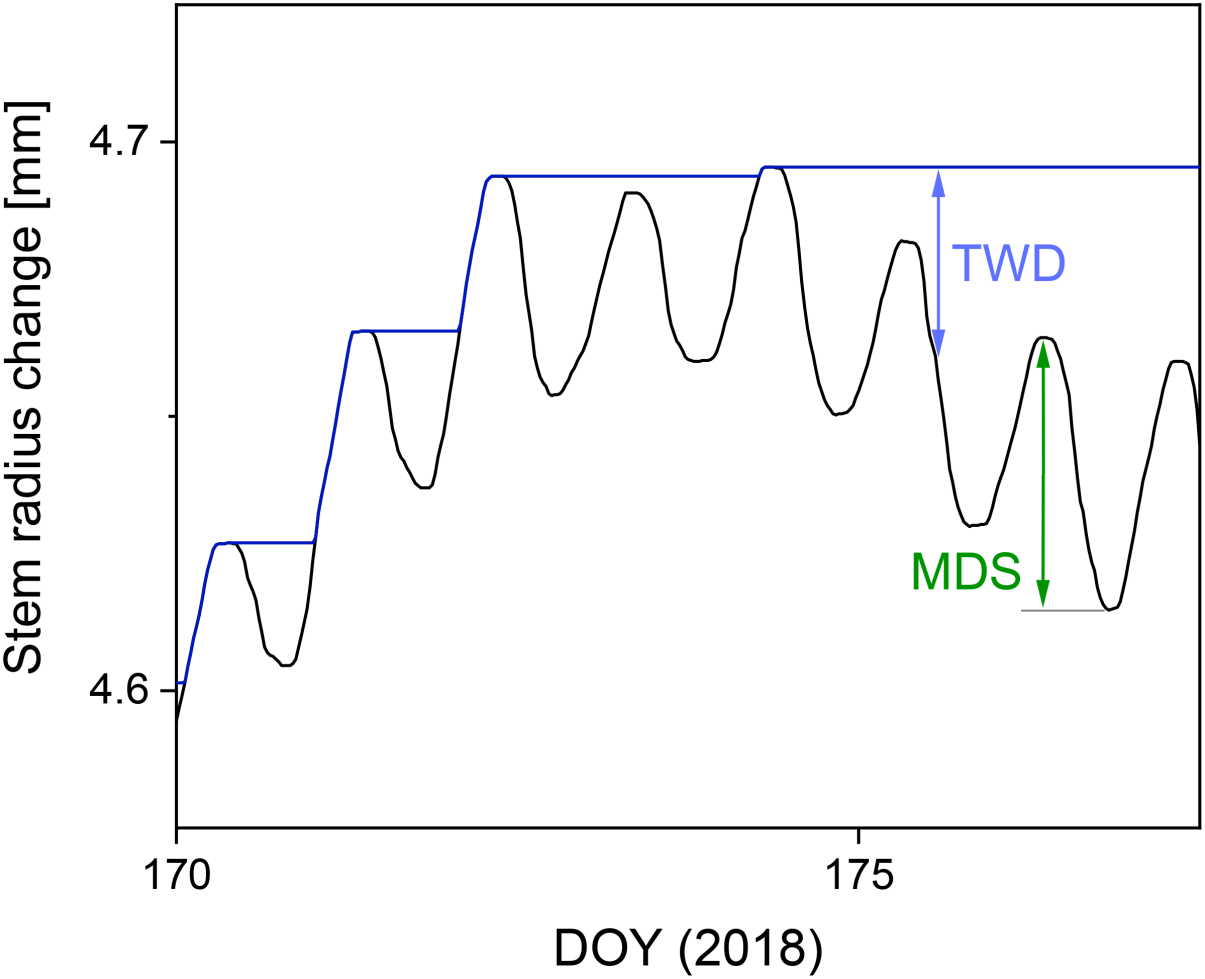
Illustration of the calculation of the water status parameters maximum daily shrinkage (MDS) and tree water deficit (TWD) based on the SRC measurements of a Douglas-fir at a clay-dominated site from June 19 to June 26, 2018. The blue line represents the last maximum SRC in accordance with the zero-growth concept (Zweifel et al., 2016).

Two water status parameters (see Section 1) were derived from SRC values for the two drought periods analyzed in summer 2018. Maximum daily shrinkage (MDS, in μm) of stem radius was calculated for each tree as the difference between the morning maximum and the subsequent minimum value (Herzog et al., 1995; King et al., 2013; Dietrich et al., 2018; Schäfer et al., 2018) (**Figure 2**). On very few days with a permanently rising stem radius, MDS was set at 0. In addition, tree water deficit (TWD, in μm) was calculated for each tree as the difference between the maximum value of the stem radius reached until time t (fully hydrated stem) and the stem radius at time t according to Zweifel et al. (2016). This assumes that no growth occurs during periods of stem shrinkage (zero growth concept) (Zweifel et al., 2016). The calculated daily values for MDS and the half-hourly calculated TWD values were averaged for each tree within one drought period (see Section 2.2).

SRC values, separately for each tree, were also the basis for estimating the days of the year (DOY) of growth onset and cessation from 2017 to 2019. Growth duration was derived for each tree by determining the number of days between growth onset and cessation. The reference base for these estimates was the total annual variation in stem radius. Growth onset and cessation were defined as reaching the 5% and 95% thresholds of total annual SRC, as used by van der Maaten et al. (2018) and Krejza et al. (2021). For these thresholds, sometimes other values were used in other studies, e.g., 2.5 or 3.5% for growth onset (van der Maaten et al., 2012; Miller et al., 2023). Since an increase in SRC in early spring may initially be caused by tissue swelling before the actual onset of growth, we used 5%. The year 2018, which was decisive for our study, showed a rather atypical course of the SRC curve. From mid/late June, many trees showed only little SRC increase and repeated periods of pronounced TWD (**Supplementary Figure 1**). We therefore used raw SRC data for the calculations of onset and cessation of growth.

### 2.5 Statistical analysis

To analyze the influence of soil and rooting characteristics on water status parameters and growth duration, we used linear mixed-effects models (LMMs). Our measurements were recorded at seven sites and were not independent from each other. LMM considers the non-independence of the data and enables the estimation of possible random effects. Model optimization was carried out using the maximum likelihood method. First, a null model (also referred to as the intercept-only model), which only contained random effects, was tested. Then, the fixed effects of the respective model equation were included, and the best-fitting model was determined using a model simplification procedure. For this model selection, the Akaike Information Criterion (AIC) was used to evaluate the relative goodness of fit of the models (Akaike, 1974). Normality and homoscedasticity were checked visually using plots of residuals vs. fitted values (Zuur et al., 2010). Possible problems with collinearity in the models were checked using the variance inflation factor (VIF) (Zuur et al., 2010). As a threshold for critical collinearity, we used VIF > 10 (Dormann et al., 2013). If a model showed high collinearity, the biological reasons for removing predictors from the model played a decisive role (Zuur et al., 2010; Harrison et al., 2018).

In Equations 1-5, water status parameters (WSP, i.e., MDS or TWD) and growth duration (GD) indicate the response variables, and *a_0_* is the overall intercept. We included the predictors soil texture (ST), PAWC, effective rooting depth (RDepth), and mean fine root density at 0–40 cm soil depth (RDens) in the LMM with Equations 1, 2, 4, 5, and thus data at site level *s*. In another model (Equation 3), we included the water status parameters MSD and TWD and thus data at tree level *t* as predictors. The coefficients *b_1_* to *b_10_* were the coefficients to be estimated of these fixed effect predictors at the site or tree level. Soil texture was a categorical variable with four levels (clay, loam, sand, silt). The other predictors were quantitative variables. The predictor soil texture was analyzed in a separate model without integrating PAWC or rooting characteristics (Equations 1, 4) because of the high collinearity between soil texture and the other variables (VIF > 10). In all LMM we used, VIF is below 10 for all predictors. In addition, possible random effects were considered. In Equations 1-5, *α* indicates the random effects at site level *s* (seven sites), and *ϵ* is the independent error term. The variances of these random effects were estimated during model fitting.

The LMM with Equation 1 was used to analyze the influence of soil texture on the water status parameters during the June drought period and the August drought period in 2018 (hypothesis 1).


(1) $$\text{WSP}=a_{0}+b_{1}{\text{ST}}_{s}+\;\alpha_{s}+\;\epsilon_{st}$$

To study the influence of PAWC and rooting characteristics on the water status parameters during the two drought periods in 2018 (hypothesis 2), we used Equation 2.


(2) $$\text{WSP}=a_{0}+b_{2}{\text{PAWC}}_{s}+b_{3}{\text{RDepth}}_{s}+b_{4}{\text{RDens}}_{s}+\;\alpha_{s}+\;\epsilon_{st}$$

The model for estimating the influence of the two water status parameters on growth duration (hypothesis 3) was as follows:


(3) $$\text{GD}=a_{0}+b_{5}{\text{MDS}}_{st}+b_{6}{\text{TWD}}_{st}+\;\alpha_{s}+\;\;\epsilon_{st}$$

To analyze the influence of soil texture, PAWC, and rooting characteristics on growth duration (hypothesis 4), we used Equations 4 , 5.


(4) $$\text{GD}=a_{0}+b_{7}{\text{ST}}_{s}+\;\alpha_{s}+\;\epsilon_{st}$$

(5) $$\text{GD}=a_{0}+b_{8}{\text{PAWC}}_{s}+b_{9}{\text{RDepth}}_{s}+b_{10}{\text{RDens}}_{s}+\;\alpha_{s}+\;\epsilon_{st}$$

**Figure 3:**
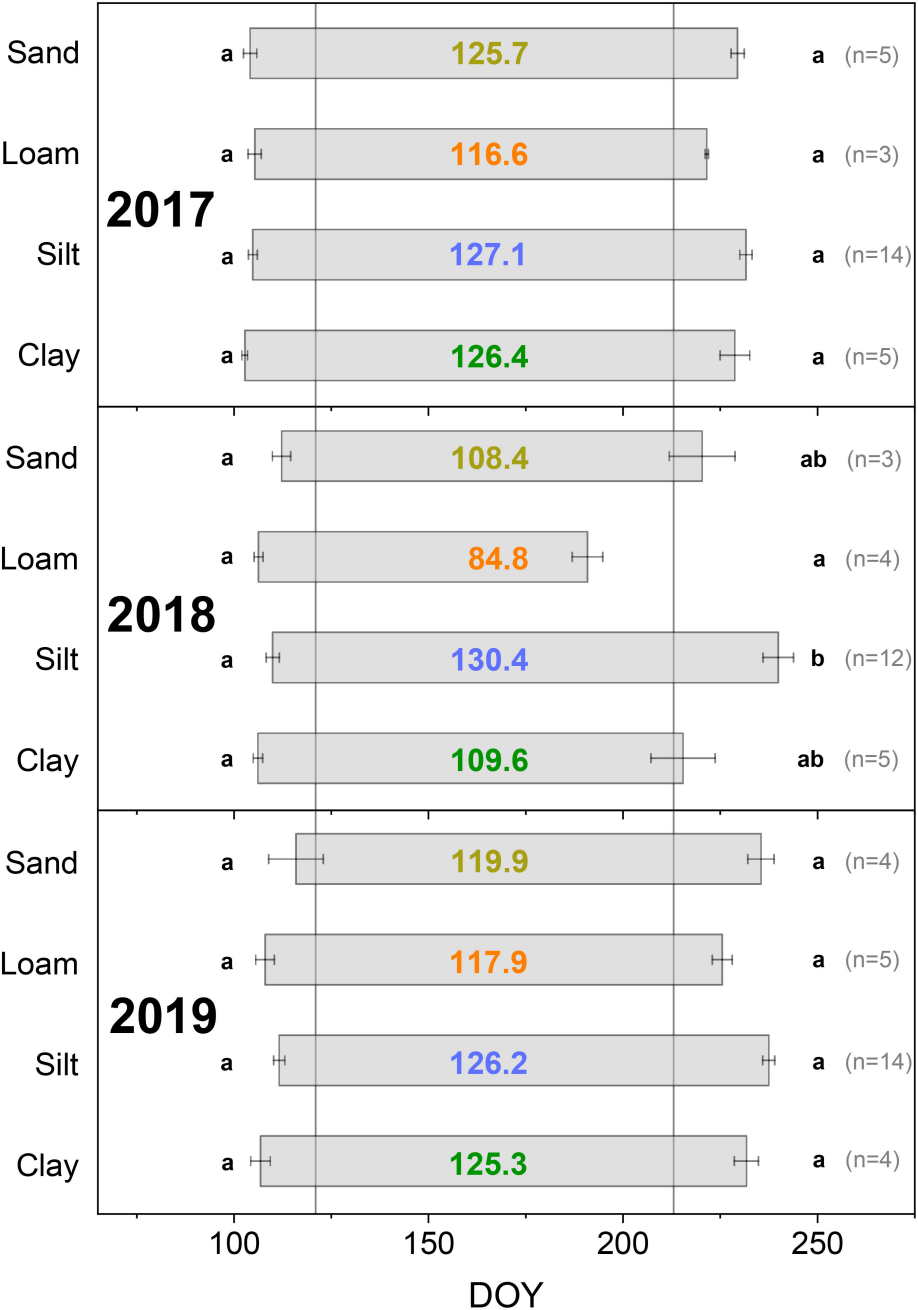
Day of growth onset and cessation depending on soil texture (determined based on dendrometer data, DOY: day of the year). Colored numbers in the bars indicate the growth duration in days. Different lowercase letters indicate a significant difference among soil textures in growth onset (left beside bars) and duration (right beside bars), *p* < 0.05. Error bars show the standard error. Vertical gray lines mark May 1 (DOY 121) and August 1 (DOY 213).

Growth onset and duration shown in **Figure 3** were tested for significant differences between several soil textures separately for each year in multiple comparisons by the Kruskal–Wallis test and a subsequent *post-hoc* test (*p* < 0.05). The *post-hoc* test used was the Dunn test. All statistical analyses were carried out using R version 4.3.2 (R Core Team, 2023). Statistical analyses using LMMs were conducted with R packages “lme4” (Bates et al., 2015), “lmerTest” (Kuznetsova et al., 2017), and “performance” (Lüdecke et al., 2021).

## 3 Results

There were no significant differences in the date of growth onset in 2017, 2018, or 2019 between the Douglas-firs in the comparison between the four soil textures within one year (**Figure 3**). Growth onset in 2018 and 2019 were similar. In 2018, this date was averaged across all soil textures at DOY 108 and in 2019 at DOY 110. In 2017, growth onset was slightly earlier (at DOY 104, see also **Supplementary Figure 1**).

There were some differences in the date of growth cessation of Douglas-firs between 2018 and quite similar years 2017 and 2019. However, the order of growth cessation was the same in all three years (loam before clay before sand before silt), even if the differences between the soil textures were much more pronounced in 2018 (**Figure 3**). Growth cessation in 2017 and 2019 differed by 3 to 6 days depending on the soil texture. In contrast, the extreme drought year 2018 led to a markedly earlier growth cessation for the soil textures loam (33 days earlier compared to the 2017/2019 average), clay (15 days earlier), and sand (12 days earlier). For the silt-dominated soils, growth cessation in 2018 occurred 5 days later than the average value for 2017/19.

### 3.1 Summer drought periods of 2018

**Figure 4:**
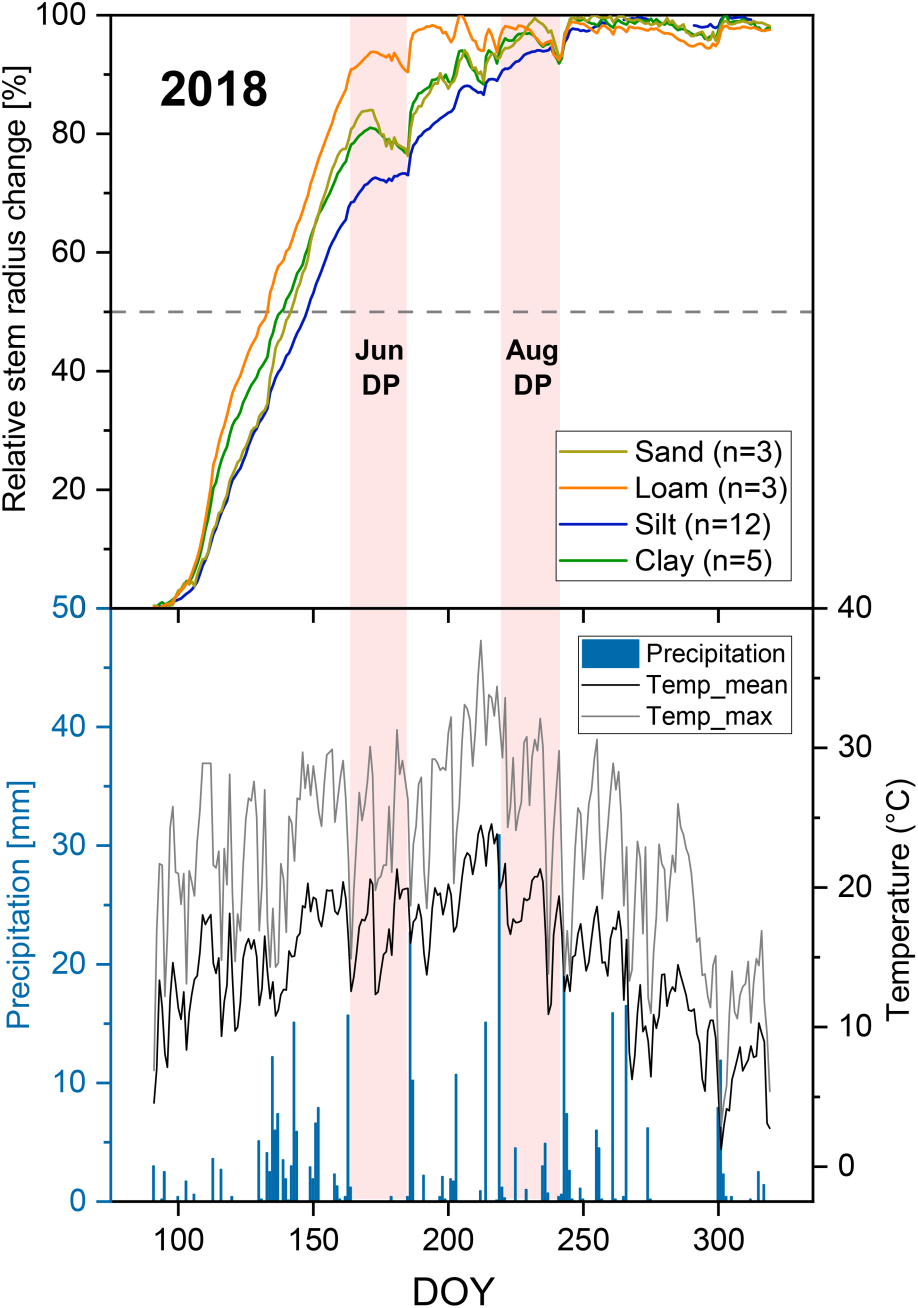
Relative stem radius change depending on soil texture (based on daily maximum values), precipitation (daily sum) and temperature (daily max and mean) in the extreme drought year of 2018. The period from April 1 to November 15 is shown. Pink bars mark the two drought periods from June 13 to July 4 (“Jun DP”) and from August 8 to August 30 (“Aug DP”) studied with a LMM (see **Figure 5** and **Tables 3**, **4**). The dashed horizontal line marks 50% of the annual growth in 2018.

**Figure 5:**
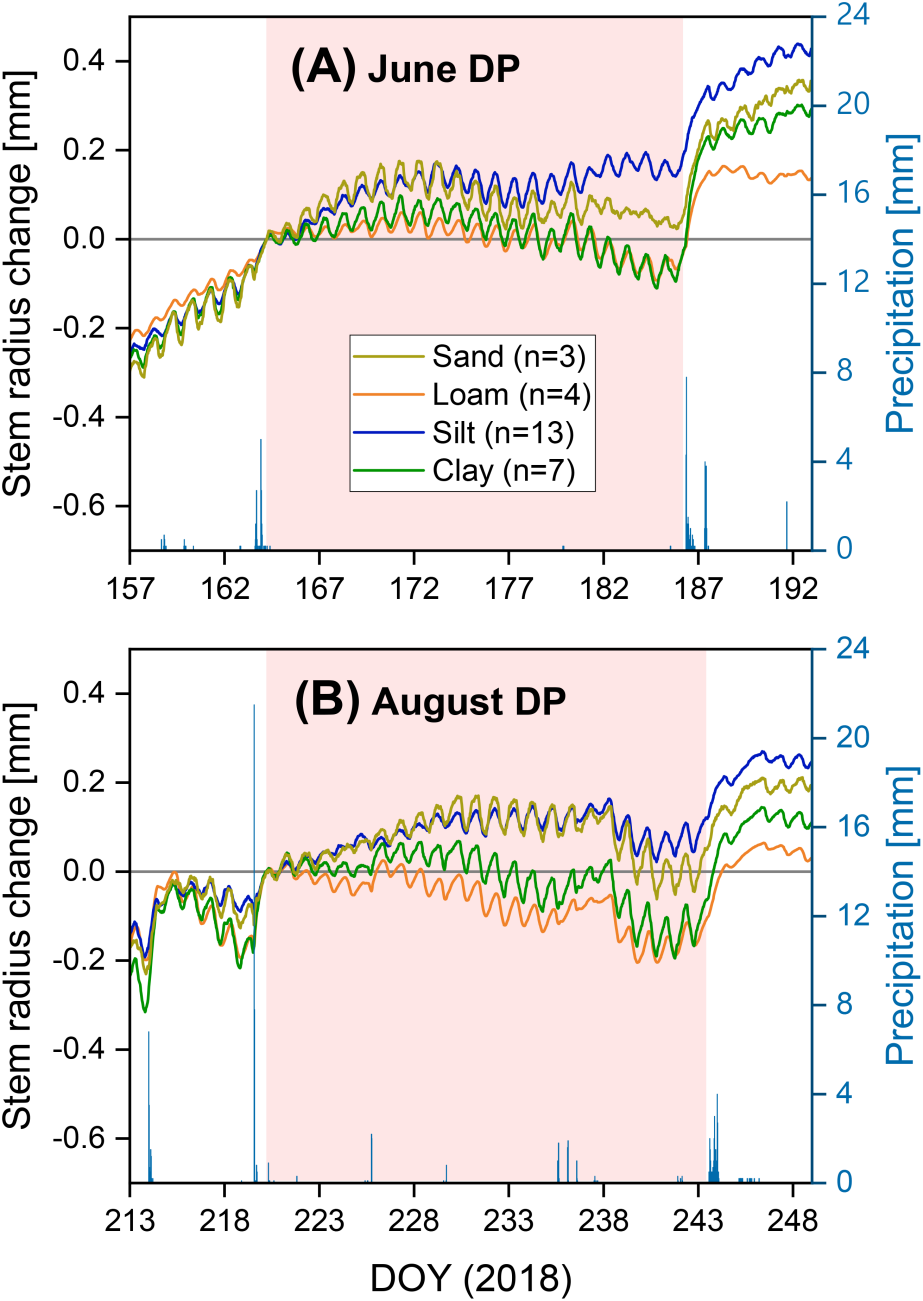
Stem radius change depending on soil texture and half-hour precipitation for two prolonged drought periods in the extreme drought year of 2018: **(A)** from June 13 to July 4 (“Jun DP”) and **(B)** from August 8 to August 30 (“Aug DP”). The curves were set to zero on the morning of the first drought day.

Both the stronger precipitation events before and after the two drought periods in 2018 and the drought periods themselves are clearly visible in the SRC diagrams for the loamy, sandy, and clayey sites (**Figures 4**, **5**). These events are less clearly recognizable in the SRC curves of the silty sites. A comparable soil texture-specific ranking was observed in both drought periods. The clay- and loam-dominated sites had lower SRC values than the silt- and sand-dominated sites, both during the drought periods and in the subsequent precipitation-induced water saturation (**Figure 5**). Up to about half to two-thirds of the drought periods, the silt- and sand-dominated sites showed a similar SRC. In the further course of the drought periods, the SRC values of the sandy site decreased slightly compared to the silty sites.

#### 3.1.1 Influence of soil texture on water status

Significant differences in the effects of soil textures were only found in the analysis of the water status parameters for TWD in the August drought period (**Supplementary Table 1**). There, the loamy site showed a significantly higher TWD than the other soil textures, while the TWD of the other three soil textures was not significantly different. The TWD of the silty and sandy sites were similar. In contrast, the clayey sites were close to the significance level (*p* = 0.083) when compared with the silty sites.

For TWD in the June drought period and for MDS in both summer drought periods, a comparatively high proportion of variance was found at the site level (**Supplementary Table 1**). In these cases, the null models showed a higher quality of fit than the models with the predictor soil texture, which showed no significant differences between the effects of soil textures in any of the cases.

#### 3.1.2 Influence of PAWC and rooting on water status

Significant influences were found mainly for the June drought period (**Table 3**). In this period, a higher PAWC reduced both TWD and MDS. A higher fine root density reduced TWD. In contrast, a higher effective rooting depth increased TWD. In the August drought period, however, there was only one significant influence; a higher effective rooting depth resulted in a reduced MDS.

**Table 3:** Results of the mixed-effects model using Equation 2: Influence of plant-available water capacity (PAWC, in mm), effective rooting depth (cm), and fine root density (n/dm^2^) on maximum daily shrinkage (MDS, in μm) and tree water deficit (TWD, in μm) during two pronounced summer drought periods in 2018 (see hypotheses 2).

Drought period June 2018		MDS		TWD	
Fixed effects		Estimate	*P*-value	Estimate	*P*-value
	*a_0_* (Intercept)	**114.922**	**<0.001**	**247.109**	**<0.001**
Predictors at site level	*b_2_* (PAWC)	**−0.774**	**0.011**	**−2.906**	**<0.001**
	*b_3_* (effective rooting depth)			**2.318**	**0.016**
	*b_4_* (fine root density)			**−9.168**	**0.010**
Random effects		Variance		Variance	
*α_s_*		31.87		0	
*ϵ_st_*		363.44		1575	
Drought period August 2018		MDS		TWD	
Fixed effects		Estimate	*P*-value	Estimate	*P*-value
	*a_0_* (Intercept)	**93.591**	**<0.001**	**90.479**	**<0.001**
Predictors at site level	*b_2_* (PAWC)				
	*b_3_* (effective rooting depth)	**−0.577**	**0.049**		
	*b_4_* (fine root density)				
Random effects		Variance		Variance	
*α_s_*		66.35		1394	
*ϵ_st_*		381.93		1879	

Data from 28 trees were evaluated. Empty cells mean that this predictor was not included in the best-fitting model. The estimated coefficients for fixed-effect predictors in the best-fit LMM and the variance of random effects are listed. Significant fixed effect estimates (*p* < 0.05) are printed in bold.

### 3.2 Growth duration 2018

Averaged across the four soil textures, growth duration was 124 days (2017), 108 days (2018), and 122 days (2019).

In both drought periods studied in 2018, a higher TWD showed a shortening of the growth duration 2018 (**Table 4**). This influence was highly significant (*p* < 0.001) in the August drought period, while the TWD influence in the June drought period was slightly above the significance level (*p* = 0.058). MDS had no significant influence on growth duration in either drought period.

**Table 4:** Results of the mixed-effects model using Equation 3: Influence of maximum daily shrinkage (MDS, in μm) and tree water deficit (TWD, in μm) on growth duration (in days) during two pronounced summer drought periods in 2018 (see hypotheses 3).

Drought period June 2018	Growth duration	
Fixed effects	Estimate	*P*-value
*a_0_* (Intercept)	**126.378**	**<0.001**
*b_5_* (MDS)		
*b_6_* (TWD)	−0.1486	0.058
Random effects		
*α_s_*	325.2	
*ϵ_st_*	203.0	
Drought period August 2018	Growth duration	
Fixed effects	Estimate	*P*-value
*a_0_* (Intercept)	**151.194**	**<0.001**
*b_5_* (MDS)	−0.205	0.148
*b_6_* (TWD)	**−0.277**	**<0.001**
Random effects	Variance	
*α_s_*	40.99	
*ϵ_st_*	141.03	

Data from 24 trees were evaluated. Empty cells mean that this predictor was not included in the best-fitting model. The estimated coefficients for fixed-effect predictors in the best-fit LMM and the variance of random effects are listed. Significant fixed effect estimates (*p* < 0.05) are printed in bold.

PAWC and the two rooting parameters had no significant influence on growth duration in the extreme drought year 2018 (**Supplementary Table 2**). In contrast, we found significant influences of soil texture on growth duration in 2018. Compared to the silt-dominated sites, the loam-, sand-, and clay-dominated sites significantly reduced the growth duration (**Table 5**, **Figure 3**). The loamy site led to the greatest reduction. Significant differences in growth duration in 2018 between Loam and Silt were also evident in the relative SRC curve (**Figure 4**). From the beginning of the first drought period on June 13, 2018, until reaching the 2018 SRC maximum, the silty sites showed 32% of the relative annual growth, while the loamy site only showed 9% (Sand: 19%, Clay: 22%). The sandy and clayey sites showed a similar relative SRC curve in 2018.

**Table 5:** Results of the mixed-effects model using Equation 4: Influence of soil texture on the growth duration (in days) in the extreme drought year of 2018 (see hypothesis 4).

2018	Growth duration	
Fixed effects	Estimate	*P*-value
*a_0_* (Intercept)	**130.391**	**<0.001**
*b_7_* (Sand)^a^	**−21.953**	**0.031**
*b_7_* (Loam)^a^	**−45.578**	**<0.001**
*b_7_* (Clay)^a^	**−20.803**	**0.015**
Random effects	Variance	
*α_s_*	0.0	
*ϵ_st_*	220.9	

Data from 24 trees were evaluated. The estimated coefficients for the respective comparisons with the reference soil texture, silt, and the variance of the random effects are listed. Significant fixed effect estimates (*p* < 0.05) are printed in bold.

a Reference is soil texture **silt.**

## 4 Discussion

In this study, we analyzed the influence of soil texture, PAWC, and two rooting parameters (effective rooting depth and fine root density in the upper 40 cm of soil) on the water status parameters TWD and MDS and on growth duration during the extreme drought year of 2018. Soil texture hardly had any significant influence on water status in both investigated summer drought periods; thus, hypothesis 1 was not confirmed. However, there were indications that silt- and sand-dominated sites produced less drought stress in the two drought periods studied. In contrast, there were significant influences of PAWC and the rooting parameters (hypothesis 2), which changed over the course of the drought in summer 2018. In the June drought period, a higher PAWC and a higher fine rooting density significantly reduced the drought stress indicator TWD. A large water reservoir and intensive topsoil rooting thus reduced drought stress in Douglas-fir in the initial period of this extreme drought. In contrast, these two characteristics had no significant influence in the later stage of the summer drought (August). However, in this August drought period, a higher effective rooting depth significantly reduced MDS, which we interpreted as drought stress reduction.

The extreme drought in 2018 led to earlier growth cessation at some soil textures and thus to a shortening of growth duration compared to 2017/2019. A greater TWD and thus greater drought stress in summer, especially in the August drought period, significantly shortened the growth duration in 2018 (hypothesis 3 is confirmed for TWD). In contrast, hypothesis 4, namely significant influence of site characteristics and rooting depth on growth duration, was not confirmed for PAWC and effective rooting depth. However, there were significant influences of the soil texture: Compared to silt-dominated soils, the sand-, clay-, and especially loam-dominated soils reduced the growth duration. In the drought year of 2018, soil texture-specific differences in growth duration were more pronounced than in 2017/2019.

### 4.1 Summer drought periods of 2018

#### 4.1.1 Silt- and sand-dominated soils resulted in higher drought tolerance

Hardly any significant differences were found in the influence of soil texture on TWD and MDS. Nevertheless, both the SRC curves (**Figure 5**) and the soil texture-specific estimates for TWD during the August drought period (**Supplementary Table 1**) indicate that Douglas-firs at silt- and sand-dominated sites perceived comparatively lower drought stress than at clayey or loamy sites. This confirms the general Douglas-fir cultivation recommendations for well-drained soils (Kutschera and Lichtenegger, 2013; Nicolescu, 2019; Thomas et al., 2022). From our results, we conclude that Douglas-fir cultivated on clayey soils showed increasing signs of drought stress during prolonged drought periods. This corresponds to a report from North America, in which young Douglas-firs on clay soils showed an increased occurrence of drought damage (Lavender and Hermann, 2014). We presume that the reason for the lower drought tolerance is a limited rooting depth on clayey soils (Köstler et al., 1968).

There are still two special aspects to be discussed: (i) The loam-dominated site showed significantly higher drought stress in August (**Supplementary Table 1**). One of the main reasons for this could be its location on the south-eastern upper slope. The resulting lower water inflow differentiates it from the other six sites. As the study based on tree ring widths at this site showed no indications of an increased drought stress risk (Spangenberg et al., 2024), loamy sites should be further investigated (see also Section 4.2). (ii) The fact that no significant differences in the effects of the four soil textures were found in the LMM analysis in the June drought period (**Supplementary Table 1**) may be due to the relatively high variance at the site level. Although the three silty sites showed no significant differences in growth duration, Silt2 and Silt3 differed markedly from Silt1 in the TWD in the June drought period.

#### 4.1.2 Drought tolerance determined by PAWC in June and by rooting depth in August

A higher PAWC reduced both TWD and MDS in the June drought period, while we did not find a significant influence on these water status parameters in the August drought period (**Table 3**). PAWC characterizes the size of the soil water reservoir available to plants (Cousin et al., 2022). A higher PAWC reduces drought stress if there is still sufficient water in the soil reservoir. In extreme drought years, even deeper soil layers severely dry out. The SMI for the total soil in **Figure 1** and the increase in TWD in August compared to June 2018 suggest that this was the case in August 2018. Our results indicate that a high PAWC reduces drought stress during moderate drought but no longer reduces drought stress in the advanced stage of an extreme drought event. This temporary effect of the soil water reservoir may explain the differing results in the literature concerning the influence of PAWC on drought tolerance in Douglas-fir (Sergent et al., 2014b; Huang et al., 2017; Spangenberg et al., 2024). However, we found a comparatively high proportion of random effects at the site level for TWD in the August drought period (**Table 3**). It is possible that the short intense rainfall events that occurred at the end of July and the beginning of August led to different surface runoff and thus to different soil infiltration. Further investigation is necessary to validate our results.

Our observation that a higher fine root density in the upper 40 cm of the soil significantly reduced TWD during the June drought period confirms our conclusions regarding the influence of PAWC. As long as there was still soil water in the upper soil layers, which was indicated by the SMI for June 2018 (**Figure 1**), intensive rooting reduced drought stress. However, if even deeper soil layers dry out, as probably happened in August 2018, a tree will not be able to extract additional water through intensive topsoil rooting. This is only possible via deeper rooting.

In our study, a higher effective rooting depth reduced MDS during the August drought. High MDS, which increased over the course of the August drought period (**Figure 5B**), indicates a large gradient between water use through transpiration and water supply from the soil, to which the tree can react due to stem water reserves (Zweifel et al., 2000; Giovannelli et al., 2007; Schäfer et al., 2018; Güney et al., 2020). In the predominantly warm to hot August drought in 2018 (**Figure 4**), Douglas-firs were exposed to the same weather conditions at all sites, and the trees at the sites with shorter tree crown lengths (Clay1 + 2, Loam, see **Table 2**) in particular showed a high MDS. Therefore, we see the explanation for the differences in MDS less in differences in water demand but primarily in differences in water supply. This supply is probably influenced mainly by different access possibilities due to the different rooting depths of the trees to the deeper soil water reserves, which are still available even when the topsoil is completely dry. When comparing diurnal SRC cycles, larger amplitudes were found in Norway spruce compared to European larch (King et al., 2013) or European beech (Schäfer et al., 2018). The authors interpreted this as faster exploitation of the internal stem water reserves by Norway spruce during a daily period. In both studies, the shallower root system of spruce is suggested as a possible explanation. Other studies on different tree species have also shown that rooting depth, root water uptake depth, and the ability to increase water uptake from deeper soil layers during drought periods have a significant influence on drought tolerance (Nardini et al., 2016; Brinkmann et al., 2019; Kahmen et al., 2022). Douglas-fir can both root deeply (Köstler et al., 1968; Thomas et al., 2015) and absorb more water from deeper soil layers during drought periods (Warren et al., 2005; Andrews et al., 2012). However, suitable soils are required for this (Köstler et al., 1968; Polomski and Kuhn, 1998). Root growth can also be influenced by certain silvicultural measures (Kühne et al., 2015; Kohnle et al., 2019; Ruge et al., 2019; Kohnle et al., 2021). Our results indicate that deep rooting can reduce drought stress in the later stage of a severe drought period, whereas high PAWC and intensive topsoil rooting are important in the first phase of severe drought.

### 4.2 Growth duration in 2018 was influenced by soil texture

For 2017, 2018, and 2019, no significant differences were found in growth onset between trees grown on different soil textures within one year (**Figure 3**). Drought year 2018 also did not cause a significant delay in growth onset in 2019 compared to 2018 for any of the soil textures. Averaged across all soil textures, the growth onset in 2019 occurred two days later than that in 2018. The SMI for the study area showed very low soil moisture until April 2019 and a recovery only from May 2019. Miller et al. (2023) found a later growth onset for Douglas-fir in 2019 compared to 2020 and saw this as the legacy effect of extreme drought in 2018. However, several studies have indicated that other factors, especially temperature, are crucial for controlling growth onset (Rossi et al., 2007; Gruber et al., 2010; King et al., 2013; Rathgeber et al., 2016; Miller et al., 2022). The influence of age and stem size on the onset of cambium division and other phases of cell differentiation found by Rossi et al. (2008) probably had no significant influence in our study, as the trees studied had a similar age, and no significant difference in growth onset was found between the different sites despite different DBH values.

The extreme drought in 2018 shortened the growth duration across all soil textures by an average of 15 days compared to 2017/19. This was primarily due to earlier growth cessation. Significant correlations between soil moisture and the date of growth cessation were found for different tree species (Gruber et al., 2010; Eilmann et al., 2011; Saderi et al., 2019; Miller et al., 2022). In our study, a higher TWD in the June drought period, but especially in the August drought period, reduced the growth duration (**Table 4**). The zero-growth concept assumes that no growth occurs during periods of stem shrinkage (Zweifel et al., 2016). Our investigations showed that even after the end of the pronounced summer drought periods, only very small radial growth occurred at some sites, resulting in a shortened growth duration as a legacy effect of a pronounced TWD.

There were, however, significant differences depending on the soil texture. The drought in 2018 significantly shortened the growth duration on the loam-, sand-, and clay-dominated sites compared to the silt-dominated sites (**Table 5**). However, resistance studies carried out based on annual increments showed different results (Spangenberg et al., 2024). There, the same silt-dominated sites showed the lowest resistance among the four soil textures and thus the greatest relative radial growth reduction in 2018 compared to the 2016/17 reference years (see also **Supplementary Figure 1**). A study by van der Maaten et al. (2018) evaluated three deciduous tree species and showed that growth duration was not a decisive determinant of the size of the annual radial stem increment. The Douglas-firs we examined showed that, in summers with more precipitation (2017 and 2019), they achieved larger radial increments even in August at certain sites (**Supplementary Figure 1**). In the extreme drought year 2018, however, a major part of annual radial growth was already reached by the middle of June (**Figure 4**). Little precipitation in July and August 2018 was not sufficient for larger radial increments. However, the plant availability for this little rainfall during drought periods varies depending on soil texture. Silt-dominated soils store a high proportion of the water available to plants and are characterized by a high PAWC (Puhlmann and von Wilpert, 2012). Clayey soils have high field capacities, but store relatively high proportions of water not available to plants (Arbeitskreis Standortskartierung, 2016). Sandy soils have a low field capacity as a large proportion of the water seeps away (Puhlmann and von Wilpert, 2012; Arbeitskreis Standortskartierung, 2016). This could explain why the Douglas-firs on the silt-dominated soils still showed radial growth at a low level in July and August and had therefore not yet reached growth cessation, while the other three soil textures showed shorter growth durations in 2018 (**Figures 3**, **4**).

As shown in Section 4.1.1, the loamy site was also noticeable in terms of growth duration. The Douglas-firs there showed the greatest absolute and relative decrease in growth duration in 2018 compared to 2017/2019 and the shortest growth duration in 2017 and 2019 (**Figure 3**). At this loamy site, which had a comparatively high PAWC, the location on the south-eastern upper slope resulted in very low water inflow, exacerbating the water shortage. As a result, the Douglas-firs there presumably had periods of drought stress also during summers with higher precipitation. The trees at this site showed the highest resistance in 2018 and thus the smallest relative decrease in tree ring width in the drought year compared with the other study sites (Spangenberg et al., 2024). This indicates an adaptation to recurring drought, e.g., in the form of a deeper rooting system. The second deepest effective rooting depth was found at this site.

Based on our results on the influences of PAWC and the two rooting parameters on TWD and MDS (Section 4.1.2), effects on growth duration—caused by effects on growth cessation—would also have been expected. However, we did not find any significant influence of these three parameters. This confirms other studies in which different or less clear influencing factors on growth cessation were found. Several factors were investigated and discussed, such as temperature, drought stress, and photoperiod (Gruber et al., 2010; Eilmann et al., 2011; Rathgeber et al., 2016; Rossi et al., 2016; Saderi et al., 2019). Their influence on growth cessation, however, is less understood than that on growth onset (Rossi et al., 2013; Rathgeber et al., 2016; Miller et al., 2022).

In the LMM with Equation 5, on which the conclusions in the previous paragraph are based, the ratio of measured trees to predictors is 8:1 and therefore less favorable than in our other LMM. To prevent such models from becoming too complex, there are different recommendations for the minimum ratio of data points to the estimated parameters (Harrison et al., 2018). Although our model with Equation 5 did not quite reach the conservative recommendation of Harrison et al. (2018) of 10:1, all reduced models with only one or two of the three predictors of Equation 5 led to comparable results.

### 4.3 Closing knowledge gaps

Studies that examine both above- and belowground biomass are important for a better understanding of the mechanisms of drought tolerance in trees. Tree roots can respond to severe drought with different strategies to avoid or tolerate drought stress (Brunner et al., 2015). These processes can also have an impact on aboveground tree growth. However, until now, there have been comparatively few studies on these complex relationships among soil, rooting, and drought tolerance on Douglas-fir. Therefore, further studies should be carried out to close existing knowledge gaps. The results of our study confirm that the rooting depth and, thus, the maximum water uptake depth is an important criterion for the possibility of stress reduction in trees during pronounced drought periods (Bréda et al., 2006).

Our study was carried out under average climatic conditions of SW-Germany on approximately 40 to 55 year old Douglas-fir trees. According to the results of the genetic analysis, the origin of the trees of all Douglas-fir stands studied is located in the central area of the natural range of the coastal variety between central Washington and northern California (Neophytou, 2019). Climatic conditions, tree age, tree species mixture, and origin of the Douglas-fir trees can influence the results (Eilmann et al., 2013; van der Maaten-Theunissen et al., 2013; Thurm et al., 2016; Vitali et al., 2017, 2018; Bréda and Brunette, 2019; Nicolescu, 2019); thus, further studies with other conditions are necessary to validate our findings. It should also be considered that our determination of growth onset and cessation is based on SRC estimates. Our results on growth duration should be verified by studies based on, for example, weekly sampling of microcores, which allow us to more precisely determine the onset and cessation of wood formation (Rossi et al., 2006; Güney et al., 2015; Rathgeber et al., 2016; Miller et al., 2022).

Despite the extreme strength of the drought in 2018, all trees studied survived and recovered well in subsequent years with higher summer precipitation in 2019 and 2020 (Spangenberg et al., 2024). There was no dieback of Douglas-fir in any of the study stands. Therefore, we do not know how the magnitudes we investigated for TWD and MDS should be assessed in comparison to the maximum possible stress level. Even within a site, there is, in some cases, a noticeable variance in magnitudes between the trees we measured. Since we examined very similar tree collectives, we interpreted this as different stress levels for individual trees. However, this can be overlaid by other tree-specific characteristics, such as different trunk and bark morphology (Brinkmann et al., 2016). Normalization based on the maximum values from the main growth period does not resolve this possible overlap. In our study, the magnitudes of TWD and MDS are the main study parameters and not onset of TWD (see Brinkmann et al., 2016). In our case, the use of normalized, i.e., relative TWD and MDS values, only brings an improvement if the reference value (e.g., a certain maximum value) can be associated with a certain stress level. This requires further studies, e.g., at the drought tolerance limit or through the parallel measurement of other stress parameters. Our approach was to investigate a comparable research question using two different methods at the same sites and trees. In addition to the dendrometer measurements described above, we also examined tree ring widths (Spangenberg et al., 2024). The conclusions from both studies fit and complement each other well. This shows that investigations using different methods can help to close knowledge gaps.

## Data Availability

The raw data supporting the conclusions of this article will be made available by the authors, without undue reservation.
