# Supplementary material for: High-resolution dendrometer measurements reveal different responses of Douglas-fir to extreme drought in 2018 depending on soil and rooting characteristics
Source: Front Plant Sci. 2024 Nov 26;15:1485440. doi: 10.3389/fpls.2024.1485440 (PMC11628273; doi:10.3389/fpls.2024.1485440)
Supplement: Supplementary file 1 [file DataSheet1.docx]

Supplementary Material

# Supplementary Tables

**Supplementary Table 1.** Results of the mixed-effects model using Eq. (1): Effect of soil texture on maximum daily shrinkage (MDS, in μm) and tree water deficit (TWD, in in μm) during two pronounced summer drought periods in 2018 (see hypothesis 1). Data from 28 trees were evaluated. Empty cells mean that this predictor was not included in the best-fitting model. The estimated coefficients for the respective comparisons with the reference soil texture, silt, and the variance of the random effects are listed. Significant fixed effect estimates (*p* < 0.05) are printed in bold.

| **Drought period June 2018** | **MDS** | | **TWD** | |
| --- | --- | --- | --- | --- |
| Fixed effects | Estimate | *P*-value | Estimate | *P*-value |
| *a_0_*  (Intercept) | **55.842** | **<0.001** | **71.355** | **0.002** |
| *b_1_* (Sand)^a^ |  |  |  |  |
| *b_1_* (Loam)^a^ |  |  |  |  |
| *b_1_* (Clay)^a^ |  |  |  |  |
| Random effects | Variance | | Variance | |
| *α_s_* | 282.6 | | 1175 | |
| *ε_st_* | 361.7 | | 1638 | |
| **Drought period August 2018** | **MDS** | | **TWD** | |
| Fixed effects | Estimate | *P*-value | Estimate | *P*-value |
| *a_0_*  (Intercept) | **62.688** | **<0.001** | **63.664** | **<0.001** |
| *b_1_* (Sand)^a^ |  |  | 5.028 | 0.848 |
| *b_1_* (Loam)^a^ |  |  | **112.877** | **<0.001** |
| *b_1_* (Clay)^a^ |  |  | 34.210 | 0.083 |
| Random effects | Variance | | Variance | |
| *α_s_* | 177.9 | | 0 | |
| *ε_st_* | 391.5 | | 1650 | |

^a^Reference is soil texture **silt**.

**Supplementary Table 2.** Results of the mixed-effects model using Eq. (5): Influence of the predictors plant-available water capacity (PAWC, in mm), effective rooting depth (cm), and fine root density (n/dm2) on the growth duration (in days) in the extreme drought year of 2018 (see hypothesis 4). Data from 24 trees were evaluated. The null model showed a higher quality of fit than the models with the predictors, which showed no significant influence on growth duration in any of the cases. Significant fixed effect estimates (p < 0.05) are printed in bold.

| **2018** | **Growth duration** | |
| --- | --- | --- |
| Fixed effects | Estimate | *P*-value |
| *a_0_*  (Intercept) | **115.915** | **<0.001** |
| *b_8_* (PAWC) |  |  |
| *b_9_* (effective rooting depth) |  |  |
| *b_10_* (fine root density) |  |  |
| Random effects | Variance | |
| *α_s_* | 248.6 | |
| *ε_st_* | 259.1 | |

# Supplementary Figures

**Supplementary Figure 1.** Mean stem radius changes of the Douglas-firs at the different study sites in the years 2017 (light blue), 2018 (red), and 2019 (dark blue). The shaded areas show standard error. Vertical gray lines mark May 1 (DOY 121) and August 1 (DOY 213).
